# Altered Arterial Stiffness, Ventricular–Arterial Coupling and Troponin Levels in Patients with Systemic Lupus Erythematosus

**DOI:** 10.3390/medicina60050821

**Published:** 2024-05-16

**Authors:** Nikolaos P. E. Kadoglou, Alexandriani Dimopoulou, Evangelia Gkougkoudi, Konstantinos Parperis

**Affiliations:** Medical School, University of Cyprus, 215/6 Old road Lefkosias-Lemesou, Aglatzia CY 2029, Nicosia 1678, Cyprus; dimopoulou.alexandriani@ucy.ac.cy (A.D.); gkougkoudi.evangelia@ucy.ac.cy (E.G.); parperis.konstantinos-marinos@ucy.ac.cy (K.P.)

**Keywords:** Systemic Lupus Erythematosus, arterial stiffness, cardio–ankle vascular index, troponin, ventricular–arterial coupling

## Abstract

*Introduction*: Systemic Lupus Erythematosus (SLE) is an autoimmune disease associated with an increased risk of cardiovascular diseases (CVDs), leading to elevated mortality rates among patients. We aimed to evaluate the levels of cardio–ankle vascular index (CAVI), global longitudinal strain (GLS), ventricular–arterial coupling (VAC), and high-sensitivity cardiac troponin I (hsTnI) in SLE patients and to explore their relationship with clinical parameters. *Methods*: This cross-sectional study enrolled 82 SLE patients without evident cardiac or kidney impairment and 41 age- and sex-matched healthy controls. We comparatively evaluated CAVI, GLS, VAC, and hsTnI between SLE patients and controls, and we assessed their association among SLE patients with disease activity based on the SELENA–SLEDAI Activity Index. Multivariate regression analysis was performed to identify independent predictors of CAVI and hsTnI within the SLE cohort. *Results*: In comparison to healthy controls, SLE patients presented with significantly higher CAVI, GLS, and hsTnI levels, while VAC was significantly reduced (*p* < 0.001). Furthermore, SLE patients with active disease (SELENA–SLEDAI ≥ 4) exhibited higher levels of CAVI and troponin than those with inactive disease (*p* < 0.001). SLEDAI was an independent predictor of CAVI, while VAC and SLEDAI were independent determinants of hsTnI in the SLE cohort. *Conclusions*: SLE patients displayed abnormal levels of CAVI, VAC, GLS, and troponin compared to healthy individuals. Our findings implicate the potential of those CV novel CVD risk factors to refine screening and therapeutic strategies for this specific population.

## 1. Introduction

Systemic Lupus Erythematosus (SLE) is a systemic autoimmune disease characterized by a wide range of clinical manifestations and an elevated risk of developing cardiovascular diseases (CVDs) compared to the age- and sex-matched general population [1]. CVD is the leading cause of mortality in SLE, attributed to accelerated atherosclerosis and altered vascular function, even at early, subclinical stages [2].

Arterial stiffness reflects changes in the mechanical properties and function of large arteries either as an essential part of atherosclerosis-prone pathophysiological mechanisms or as a consequence of CVD [3]. Its independent association with increased cardiovascular risk is widely known and its assay could assist the CVD stratification in a wide range of populations. Pulse wave velocity (PWV) constitutes a non-invasive, simple measurement of arterial stiffness, already measured and validated in a plethora of studies. It has been recognized as a marker of vascular aging and has been found elevated in early stages of CVD development or in high-risk patients (e.g., diabetes) [4]. Thereby, scientific societies (the European Society of Cardiology and the European Society of Hypertension) recommend measuring of PWV as a screening test in the general population or as a surrogate index of CVDs, advocating its routine clinical application in cardiology daily practice [5,6,7]. In the context of SLE, characterized by higher CV risk, a recent meta-analysis of nine studies found significantly higher PWV in SLE patients compared to controls [8]. However, the measurement of PWV is notably susceptible to blood pressure variations, posing a significant limitation. To overcome it, another formula of PWV calculation, termed cardio–ankle vascular index (CAVI) has been proposed [9]. Previous studies have demonstrated the discriminatory power of CAVI to stratify the CVD risk in patients without previous overt CVDs [10]. Despite the increasing use of CAVI in other patient populations, its clinical application in SLE cohorts remains unexplored, indicating a significant gap in research.

The role of myocardial strain and the ventricular–arterial coupling (VAC) in the pathophysiology of CVDs is well established [11]. The former can detect even subtle myocardial dysfunction in a wide spectrum of cardiac and non-cardiac conditions. A previous study has reported a marked increase in global longitudinal strain (GLS), an index of myocardial strain, in SLE patients even without apparent cardiac disease [12]. Besides this, the vascular injury and increased arterial stiffness in SLE patients seems to be a rational explanation of the frequent cardiovascular complications recorded in SLE patients [13]. The interplay between cardiac dysfunction and increased afterload due to arterial stiffening is well depicted by VAC estimation. Several parameters have been proposed for VAC calculation [14]. Recently, the PWV/GLS ratio has been proposed as a reliable, feasible, easily performed, and reproducible index of VAC with prognostic value [15]. However, very scarce data exist about the impact of SLE on VAC [16]. From the clinical perspective, the early identification of cardiovascular dysfunction with impaired VAC could stratify the cardiovascular risk and determine the therapeutic management of SLE.

The detection of biomarkers of myocardial injury, such as troponin, has been raised as an additional way to assess the cardiac dysfunction in SLE patients. A recently published study demonstrated that women with SLE, normal kidney function, and increased PWV were more likely to have detectable levels of high-sensitivity troponin (hsTn) [17]. This finding suggests that hsTn could help stratify the risk of atherosclerotic lesions’ presence in SLE patients [18]. Emerging evidence points to elevated troponin levels in SLE patients as indicative of subclinical cardiac involvement, occurring even in the absence of overt cardiac complications such as myocarditis or acute myocardial infarction [19].

The objective of the present study was to conduct a comparative analysis of the CAVI, VAC, and hsTnI between SLE patients and healthy controls. In addition, we assessed the relationship of CAVI and troponin with disease activity and other clinical parameters.

## 2. Materials and Methods

### 2.1. Study Design

This is an observational, single-center, cross-sectional study enrolling 82 patients diagnosed with SLE according to SLICC 2012 classification criteria [20]. Part of our data of this cohort have been submitted elsewhere and they are under peer-reviewing for publication. Any possible SLE-related manifestations were retrieved from medical records. We excluded SLE patients with concomitant established CVD. In particular, patients with unambiguously diagnosed or suspected coronary artery disease (CAD), heart failure, peripheral artery disease (PAD), or other cardiomyopathies were excluded. The SLE disease activity was examined using the Safety of Estrogens in Lupus Erythematosus National Assessment–SLEDAI instrument score (SELENA–SLEDAI). The SELENA–SLEDAI is a validated, comprehensive 24-item instrument designed to assess SLE disease activity over the preceding 10 days. It encompasses the evaluation of clinical signs and symptoms, laboratory findings, and specific conditions noted during the patient’s visit [21].

A group of 41 age- and sex-matched healthy subjects with no chronic disease served as control group. In particular, the control group consisted of individuals without any cardiovascular, systemic inflammatory or autoimmune conditions, hypertension, diabetes, kidney or liver dysfunction, and atrial fibrillation. Subjects who had experienced acute inflammatory decompensation, systematic infections, or surgeries in the past month were also excluded from this study. This study adhered to the guidelines of the Declaration of Helsinki and was approved by the national bioethical committee (Reference number of approval: EEBK/EEP/2021/34). Before entering this study, all participants provided a signed informed consent.

### 2.2. Participants’ Clinical Examination

During the clinical examination of participants, a structured questionnaire was used to document medications and comorbidities. This study defined hypertension as the use of antihypertensive drugs or blood pressure readings ≥ 140/90 mmHg; dyslipidemia as the use of hypolipidemic drugs or LDL cholesterol levels ≥ 130 mg/dL; active smoking as current smoking or cessation within the last 6 months; diabetes mellitus as the use of antidiabetic drugs, fasting plasma glucose ≥ 126 mg/dL, or HbA1c ≥ 6%; and coronary artery disease as a history of angina, myocardial infarction, or myocardial revascularization. Blood pressure was measured twice in a sitting position after a 5 min rest, and participants’ weight was recorded to calculate BMI.

### 2.3. Arterial Stiffness Assessment

To measure the arterial stiffness of the participants, the CAVI method was used (Vasera VS-1500, Fukuda Denshi, Tokyo, Japan). This is a non-invasive and reproducible technique independent of blood pressure during the time of measurement. CAVI is calculated by the following formula: CAVI = a {(2ρ/∆P) × ln (SBP/DBP) PWV2} + b (a and b are constants, PWV: pulse wave velocity from the heart to the ankle, SBP: systolic blood pressure, DBP: diastolic blood pressure). CAVI is expressed in arbitrary units and has a normal value of less than 9. Higher values indicate increased arterial stiffness and a higher risk of CVDs. We followed the standard procedure recommended by the manufacturer already described in our previous study [22]. All participants rested in a supine position for at least 10 min. The ECG electrodes were placed on both wrists, a phonograph for detecting heart sounds was placed at the base of the sternum, and 4 cuffs were wrapped around both the arms and ankles. CAVI can be calculated from PWV at the origin of the aorta to the distal segment of tibial artery (ankle), and the systolic and diastolic blood pressures can be measured at the brachial artery. After entering the height, weight, age, and gender, the device automatically calculates the right and left CAVI by measuring the transit time of pulse wave to each ankle and we then calculate the mean value. Furthermore, the device provided ankle–brachial index (ABI) for both sides [23,24,25].

### 2.4. Global Longitudinal Stain (GLS) 

Global longitudinal stain (GLS) was used to calculate the LV myocardial deformation. During breath-holding, longitudinal strain was measured from the 3 apical views and each wall was subsequently divided into 3 segments (basal, mid, and apical) and a total of 17 segmental strain curves was obtained, using the EchoPAC Version 203 software package (GE Vingmed Ultrasound, Horten, Norway). The frame rate frequency was >60 frames/s. GLS was calculated as the average value of the three apical strain peak values. The intra- and inter-observer reliability of strain analysis by our group has been previously reported and it is very low (<2.5%) [26]. 

### 2.5. Blood Assays

Blood samples were collected, immediately centrifuged, and the serum was stored at −80 °C for preservation. Serum high-sensitivity troponin I (hsTn I) measurement was performed using the Alinity analyzer (Abbott Diagnostics, Abbott Park, IL, USA). The normal range is <34.5 pg/mL for men and <15.6 pg/mL for women. This is a two-step immunoassay in human serum. This is based on chemiluminescent microparticle immunoassay (CMIA) technology, which combines and incubates the sample and paramagnetic microparticles coated with antitroponin I antibodies (anti-cTnI Ab). According to the manufacturer, the precision of the hs Tn I assay at low concentrations is sufficient to enable the assessment of a range of thresholds with 3.2% CV of our lab [27]. 

### 2.6. Statistical Analysis

Normally distributed continuous variables were presented as the mean ± SD. Normality of distribution was assessed by the Kolmogorov–Smirnov test. Comparisons of continuous and categorical variables were analyzed with the student’s *t*-test and chi-square test, respectively. Changes of continuous variables within groups were assessed using paired samples *t*-test. To test the univariate associations of either CAVI or hsTn I with any of the study population characteristics, we performed a Pearson correlation. Variables with normal distribution and significant correlation entered the multiple linear regression analysis models. A two-tailed *p* < 0.05 was considered as significant. The computer software package SPSS (version 25.0; SPSS Inc., Chicago, IL, USA) was used for statistical analysis.

## 3. Results

### 3.1. General Characteristics

In this study, 82 SLE patients and 41 age- and sex-matched healthy controls (2:1 ratio) were enrolled, with a high female representation in both groups (89% and 90%, respectively) (Table 1). Regarding SLE-related medications, 38% were on oral treatment with prednisone. The vast majority was receiving 2.5–10 mg daily. Moreover, more than 80% of the SLE patients were on hydroxychloroquine (200 mg once daily). Within the SLE group, approximately 30% of the patients had hypertension, 28% dyslipidemia, and a very small percentage (2%) had diabetes. Moreover, 21 patients had experienced nephritis and another 11 patients reported pericarditis in the past. None of them had impaired kidney function nor peripheral arterial disease as expressed by the ankle–brachial index (ABI). There were no significant differences in clinical parameters between groups (*p* > 0.05). Compared with the control group, SLE patients had significantly higher levels of CAVI, hsTn I, GLS, and lower VAC values (*p* < 0.001). All those results are presented in Table 1. Notably, we compared SLE patients without hypertension or hyperlipidemia with controls and the significant differences in CAVI, hsTn I, GLS, and VAC between groups remained unaltered, implicating that those co-morbidities did not influence those results.

Based on the disease activity (SLEDAI), we further examined the differences between subgroups of the SLE cohort: 

Subgroup A (N = 46): Patients with low-activity disease (SLEDAI < 4).

Subgroup B (N = 36): Patients with moderate- or high-active disease (SLEDAI ≥ 4). 

Although those subgroups did not differ in their clinical characteristics, subgroup B exhibited higher levels of CAVI (*p* = 0.002) and troponin (*p* = 0.016) than subgroup A. No significant differences were observed in the rest of the parameters (Table 2).

### 3.2. Correlations

In the SLE group, CAVI showed significant correlations with age (*p* < 0.001), SLEDAI scores (*p* < 0.001), and troponin (*p* = 0.029). Those variables entered the multiple regression analysis model and after adjustment for age, SLEDAI was an independent determinant of CAVI (R^2^ = 0.485. *p* < 0.001) (Table 3). 

In addition to CAVI, troponin levels were associated with age (*p* = 0.019), CAVI (*p* = 0.029), SLEDAI (*p* = 0.006), and VAC (*p* < 0.001). In multiple regression analysis, VAC and SLEDAI remained independent determinants of troponin levels (Table 4). On the other hand, we failed to find any other independent relationship of either GLS or VAC with the rest of the clinical parameters.

## 4. Discussion

In the present study, we comparatively evaluated arterial stiffness, GLS, VAC, and troponin levels between SLE patients and healthy subjects. We observed increased CAVI, GLS, and troponin and lower VAC levels in SLE patients compared to controls. Those significant differences remained after excluding from analysis patients with classical cardiovascular risk factors, such as hypertension or hyperlipidemia. Moreover, SLE patients with at least moderate disease activity had even greater CAVI and troponin levels than their counterparts with low disease activity. Notably, SELENA–SLEDAI ≥ 4, a measure of at least moderate disease activity, was found to independently predict CAVI and troponin levels, emphasizing the direct impact of SLE activity on these cardiovascular markers. 

SLE may lead to subtle changes in cardiac function, where early detection could substantially mitigate the irreversible cardiovascular system damage. The measurement of arterial stiffness in the SLE population is associated with a higher cardiovascular risk [28]. Most but not all observational studies have reported higher arterial stiffness than controls [29,30], using the classical carotid-femoral probes for PWV calculation, with its inherent limitations. In particular, the classical assessment of PWV with the probe’s simultaneous placement at the carotid and femoral arteries is blood pressure-dependent and it is also affected by mistakes in distance calculation and the probe’s stability during measurements. To our knowledge, this is the first study evaluating arterial stiffness in SLE patients using the CAVI technique. Our study showed that SLE patients had significantly higher CAVI levels than controls, demonstrating CAVI’s utility in assessing arterial stiffness without the influence of blood pressure on those measurements. Prior research has highlighted the role of age, mean arterial pressure, renal function, and various comorbidities on PWV among SLE patients [8,31]. SLE is usually complicated with CVD and therefore increased arterial stiffness may be the late consequence of previous complications and/or co-existing CVD. In our study, SLE patients were CVD-free regarding CAD, heart failure, and PAD. Previous nephritis or pericarditis did not confer overt changes in kidney and cardiac function, respectively. Despite the absence of obvious cardiovascular complications, our findings further emphasize that active SLE disease is associated with CAVI elevation, and their interplay seems independent of cardiovascular complications. This is of clinical importance outlining that a high cardiovascular risk persists even in our CVD-free SLE population, which has been reported in previous studies [28,32]. Regarding the prognostic utility of CAVI in SLE patients, its employment for cardiovascular risk stratification could revolutionize patients’ management by endorsing more aggressive therapeutic approaches in otherwise uncomplicated SLE patients. Numerous studies have demonstrated the association of CAVI with the development of cardiovascular events in patients with established atherosclerotic CVDs (ASCVDs) [33,34] or those at high risk for ASCVDs [35]. No data are available for the prognostic value of CAVI in the SLE population and this remains to be proved. 

In agreement with previous studies, we confirmed the significant elevation of GLS in our SLE patients. A number of studies have previously reported the association of GLS with SLE, even in the absence of overt cardiac dysfunction [36]. Such an observation has convinced some investigators to recommend the application of the speckle tracking technique for the diagnosis of cardiac complications (e.g., myocarditis) or cardiac involvement of SLE patients at an early stage [37]. Regarding the complex interplay between cardiac and vascular function in SLE patients, we hypothesized that VAC assessment may be superior to GLS or arterial stiffness as the sole measurement [38]. While most related studies have focused on the diagnostic and prognostic value of VAC in hypertensive patients [39], the role of VAC in evaluating the effects of novel anti-hypertensive and anti-diabetic treatments has also been noted [40]. In our cohort, the calculated CAVI/GLS ratio, as a measure of VAC showed significantly lower values in SLE than controls, mainly driven by the elevated CAVI levels. The presence of at least moderate-active disease was associated with further decline in VAC. Up to now, very scarce data exist about the impact of autoimmune diseases on VAC [41]. A brief report of a small group of female patients with SLE showed a considerably increased PWV/GLS ratio compared to controls [10]. This is the second study in the SLE population reporting a declined VAC which could be of clinical relevance, since VAC has the potential to detect early organ damage in SLE and evaluate medication efficacy, which is promising. This is further supported by the independent association of VAC with hsTn levels. Finally, prospective studies are needed to confirm this hypothesis, and the formula of the CAVI/GLS ratio for VAC assessment requires further validation.

HsTn is a well-known biomarker of myocardial injury. After excluding obvious acute, subacute, or chronic cardiac complications, like myocarditis or myocardial infarction, hsTn may be found elevated in SLE implicating most usually cardiac involvement or other extra-cardiac causes which was difficult to identify and rule out in our study [16]. In agreement with recent research, our study verified increased hsTn levels in SLE patients characterized by a lack of apparent kidney or cardiac dysfunction and a low CV risk profile [14]. Compared to that study, which utilized PWV and categorized hsTn and PWV as dichotomous variables, our study employed CAVI to assess arterial stiffness and analyzed both hsTn and CAVI as continuous variables. This approach revealed a significant, independent association between hsTn levels, CAVI, and VAC, suggesting a link between subtle myocardial injury in SLE and functional alterations in cardiovascular function including large arteries. Although our study could not establish a direct causal link, the significant impact of SELENA–SLEDAI on hsTn levels highlights a complex relationship between inflammatory processes, myocardial injury, and arterial stiffness in SLE patients. Winau L. et al. (2018) reported inflammatory interstitial remodeling and edema as the possible causes of hsTn elevation and subclinical myocardial injury, detectable by cardiac magnetic resonance (CMR) [42]. The clinical significance of even a slight rise in hsTn is well recognized across various diseases, emphasizing its utility as a highly sensitive marker for cardiac diseases [43,44]. Therefore, the elevated values and the interplay of those two potent CV predictors (CAVI and hsTn) in the SLE population predispose patients to an adverse prognosis. In a recent large, randomized, double-blind, placebo-controlled, multicenter trial, hsTn predicted the incidence of CV events during a follow-up period of over 20 months among SLE patients without a history of CVD [45]. However, more data are required to confirm this hypothesis and implement the regular use of hsTn in the SLE population, especially in those without overt CVD.

There are several limitations in the current study. The relatively small sample size of SLE patients without CVD, with mostly low or moderate disease activity and the limited number of CV risk factors might have impeded our ability to detect differences in GLS or identify independent associations with demographic and clinical variables in patients with more active disease. Moreover, the cross-sectional design of our study did not allow us to draw firm conclusions about the predictive value of CAVI for cardiovascular events in SLE patients, as well as the potential role of treatment in modifying CAVI measurements. The addition of CMR examination in our study would have provided more comprehensive information about cardiac involvement. Additionally, the possibility of mild chronic myocarditis cannot be definitively ruled out. Lastly, as our study was conducted at a single center and predominantly included female and Caucasian patients, the generalizability of the findings to broader populations remains uncertain. 

## 5. Conclusions

In conclusion, SLE patients without cardiac or kidney impairment had abnormal values of CAVI, GLS, VAC, and hsTn I levels compared to healthy controls. Notably, at least moderately active disease further prompted CAVI and troponin elevation, suggesting cardiac involvement even in SLE patients without apparent cardiac manifestations. Our findings have the potential to stratify the CV risk in SLE patients and their clinical application may refine the screening tests and therapeutic approach of this specific cohort.

## Figures and Tables

**Table 1:** Comparison between SLE patients and healthy controls.

	SLE Group N = 82	Control Group N = 41	*p*-Value
**Age (years)**	50 ± 15	49 ± 7	0.455
**Males/females (n)**	9/73	4/37	0.990
**Hypertension (n)**	24 (29%)	0	-
**Dyslipidemia (n)**	23 (28%)	0	-
**Diabetes (n)**	2 (2%)	0	-
**Duration SLE (years)**	15 ± 6	-	-
**SLEDAI**	2.91 ± 3.15	-	-
**BMI (Kg/m^2^)**	24.92 ± 4.63	26.46 ± 4.99	0.076
**SBP (mmHg)**	134 ± 16	130 ± 13	0.175
**DBP (mmHg)**	82 ± 11	82 ± 8	0.989
**LVEF (%)**	65 ± 7	67 ± 6	0.215
**RABI**	1.00 ± 0.15	1.02 ± 0.09	0.495
**LABI**	1.02 ± 0.10	1.03 ± 0.11	0.948
**GLS (%)**	−19.92 ± 2.70	−21.15 ± 1.55	<0.001
**hsTn I (ng/mL)**	3.27 ± 2.25	1.56 ± 1.12	<0.001
**CAVI**	7.60 ± 1.26	6.79 ± 0.86	<0.001
**VAC (CAVI/GLS ratio)**	−0.39 ± 0.95	−0.32 ± 0.48	<0.001

Abbreviations: BMI, Body–mass index; CAVI, Cardio–ankle vascular index; DBP, Diastolic blood pressure; GLS, Global longitudinal strain; hsTn I, High-sensitivity troponin I; LABI, Left ankle brachial index; LVEF, Left ventricular ejection fraction; n, number; RABI, right ankle brachial index; SBP, Systolic blood pressure; SLE, Systematic lupus erythematosus; SLEDAI, Systemic Lupus Erythematosus Disease Activity Index; VAC, ventricular–arterial coupling.

**Table 2:** Comparison between SLE patients with at least moderate disease activity (SLEDAI ≥ 4) (subgroup A) and SLE patients with low disease activity (SLEDAI < 4) (subgroup B).

	Subgroup A (SLEDAI < 4) N = 46	Subgroup B (SLEDAI ≥ 4) N = 36	*p*-Value
**Age (years)**	50 ± 15	50 ± 14	0.792
**Hypertension (n)**	13 (28%)	11 (31%)	0.881
**Dyslipidemia (n)**	11 (24%)	13 (36%)	0.191
**Diabetes (n)**	1	1	-
**Nephritis (n)**	11 (23.9%)	10 (28%)	0.332
**Pericarditis (n)**	5 (11%)	6 (16.7%)	0.127
**Duration SLE (years)**	15 ± 6	17 ± 8	0.812
**BMI (Kg/m^2^)**	24.17 ± 4.37	25.88 ± 4.86	0.098
**SBP (mmHg)**	137 ± 15	132 ± 17	0.141
**DBP (mmHg)**	82 ± 12	82 ± 11	0.980
**LVEF (%)**	64 ± 7	63 ± 7	0.624
**RABI**	1.01 ± 0.12	1.01 ± 0.09	0.859
**LABI**	1.02 ± 0.10	1.03 ± 0.11	0.948
**GLS (%)**	−20.07 ± 2.76	−19.74 ± 2.64	0.584
**Troponin (pg/mL)**	2.74 ± 2.07	3.94 ± 2.32	0.016
**CAVI**	7.40 ± 1.34	8.06 ± 1.13	0.002
**VAC (CAVI/GLS ratio)**	−0.37 ± 0.10	−0.40 ± 0.07	0.200

Abbreviations: BMI, Body–mass index; CAVI, Cardio–ankle vascular index; DBP, Diastolic blood pressure; GLS, Global longitudinal strain; LABI, Left ankle brachial index; LVEF, Left ventricular ejection fraction; n, Number; RABI, Right ankle brachial index; SBP, Systolic blood pressure; VAC, ventricular–arterial coupling.

**Table 3:** Associations between CAVI (dependent variable) and other variables within SLE patients.

Variables	Univariate Analysis		Multivariate Analysis	
	β (SE)	*p*	β (SE)	*p*
Age	0.677 (0.434)	<0.001	0.543 (0.337)	<0.001
Troponin	0.385 (0.532)	0.029		
SLEDAI	0.547 (0.321)	<0.001	0.210 (0.198)	0.012

Abbreviations: SLEDAI, Systematic Lupus Erythematosus Disease Activity Index.

**Table 4:** Associations between troponin and other variables within SLE patients.

Variables	Univariate Analysis		Multivariate Analysis	
	β (SE)	*p*	β (SE)	*p*
Age	0.589 (0.610)	0.019		
CAVI	0.385 (0.532)	0.029		
SLEDAI	0.303 (0.201)	0.006	0.208 (0.158)	0.041
VAC	0.426 (0.247)	<0.001	0.281 (0.145)	0.012

Abbreviations: CAVI, Cardio–ankle vascular index; SLEDAI, Systematic Lupus Erythematosus Disease Activity Index; VAC, Ventricular–arterial coupling.

## Data Availability

Data are contained within the article.
